# Incidence of Fish-mouthing and Wall Malapposition of Different Flow Diverters in the Treatment of Cerebral Aneurysms: Device-specific Findings in a Retrospective Single-center Analysis

**DOI:** 10.1007/s00062-026-01651-7

**Published:** 2026-04-15

**Authors:** Maximilian Rothe, Martin Renz, Maria Berndt-Mück, Dennis Hedderich, Dominik Sepp, Bernhard Meyer, Chiara Negwer, Jannis Bodden, Jan Kirschke, Tobias Boeckh-Behrens, Christian Maegerlein

**Affiliations:** 1 Institute of Neuroradiology, Technical University of Munich, School of Medicine and Health, Munich, Germany; 2 Department of Neurosurgery, Technical University of Munich, School of Medicine and Health, Munich, Germany

**Keywords:** Flow Diverter, Fish-mouthing, Wall apposition, DERIVO embolization device, Braid stability, Intracranial aneurysm

## Abstract

**Purpose:**

Flow diverter (FD) braid stability is crucial for optimal wall apposition, thereby reducing the risk of device-related complications following intracranial aneurysm treatment. We compared wall apposition characteristics across different FD designs to evaluate inter-device differences.

**Methods:**

Retrospective single-center analysis of 104 patients with 127 aneurysms treated with 121 FD (35 Derivo Embolization Devices (DED; Acandis, Pforzheim, Germany), 50 p64 (Phenox Wallaby, Bochum, Germany), 24 p48 (Phenox), 11 Silk (Balt, Montmorency, France), and 1 Pipeline (Medtronic, Dublin, Ireland)) between February 2013 and June 2023. The analysis focused on fish-mouthing-like deformities (≥ 10% focal diameter reduction at device ends) and the need for mechanical wall apposition maneuvers, as well as aneurysm occlusion, thrombus-associated events, and neurological outcomes.

**Results:**

Fish-mouthing-like deformities occurred more frequently with DED (50.0%) than with other FD-types (27.2%; *p* = 0.021), mainly due to “pre”-fish-mouthing (31.3% vs. 14.8%; *p* = 0.047). Mechanical wall apposition maneuvers were more often required in DED cases (60.0% vs. 25.6%; *p* < 0.001), particularly for proximal malapposition. Aneurysm occlusion rates were comparable at final FU (84.2% vs. 88.6%; *p* = 0.563). Thrombus-associated events occurred more frequently with DED (29.4% vs. 11.3%; *p* = 0.008), though neurological outcomes did not differ significantly (6.0% vs. 2.6%; *p* = 0.148). Mean FU time was 26.0 ± 21.0 months.

**Conclusion:**

DED required more frequent intraprocedural wall apposition maneuvers and showed higher rates of fish-mouthing-like deformities compared with other FD-types. However, long-term aneurysm occlusion and neurological outcomes were comparable. These findings suggest that while DED deployment may require greater technical expertise, its safety and efficacy should be further evaluated in larger, multicenter studies.

**Supplementary Information:**

The online version contains supplementary material available at (https://doi.org/10.1007/s00062-026-01651-7).

## Introduction

Flow diversion has transformed aneurysm therapy since the first clinical use of the Pipeline Embolization Device (PED; Medtronic, Dublin, Ireland) in 2008. Initially restricted to large, wide-necked aneurysms of the internal carotid artery (ICA) indications have expanded to smaller and more complex lesions as operator experience and device technology have advanced [1].

Despite excellent occlusion rates exceeding 80% in most series [2], device-related complications, such as thromboembolic events and wall malapposition remain relevant concerns [3]. Among structural braid instabilities, *fish-mouthing*—a focal narrowing at one or both device ends [4]—has gained attention for its potential to cause hemodynamic disturbance or serve as a thrombogenic nidus [5]. The reported incidence of fish-mouthing varies widely (0.75 to 36%) [6, 7], reflecting differences in devices, imaging protocols, and definitions [4]. Risk factors include inadequate wall apposition [8], oversizing [9], vessel tortuosity [8], and mechanical design characteristics such as wire stiffness, braid angle, and material composition, including the use of drawn-filled tubing (DFT) wires technology in some devices [10]. Over the past decade, multiple second- and third-generation flow diverters (FDs) have been introduced, differing in braid density, wire architecture, alloy composition, and surface coatings. These refinements aim to enhance deliverability, wall apposition, and hemocompatibility, yet they also introduce variability in mechanical behavior that may influence in-vivo performance and deformation patterns [11]. Comparative clinical data on fish-mouthing across device platforms remains limited, and real-world observations often precede systematic evaluation [8].

The Derivo Embolization Device (DED; Acandis, Pforzheim, Germany) is a self-expanding, DFT-based FD with flared ends. During the 2025 annual meeting of the Berufsverband Deutscher Neuroradiologen e. V. (BDNR), colleagues from several centers had reported an apparent increase in fish-mouthing-like deformities with this device. Motivated by these early observations—and against the background of our own generally favorable experience with the DED—we conducted a retrospective single-center analysis comparing DEDs with other FD-types. This exploratory study aims to provide initial data on device braid stability and wall apposition performance, while emphasizing the need for larger, preferably prospective multicenter investigations.

## Patients and Methods

### Study Design

This retrospective single-center study followed STROBE guidelines [12]. Consecutive patients undergoing FD implantation between February 2013 and June 2023 were screened (*n* = 160). Inclusion criteria were: (1) availability of pre-procedural 3D rotational angiography (3DRA) with stored datasets, (2) deployment of ≤ 2 FD per aneurysm, and (3) adequate imaging and clinical follow-up (FU) for outcome assessment. Both ruptured (*n* = 11) and unruptured (*n* = 116) aneurysms were included. The reasons for exclusion comprised unavailability of 3DRA datasets (*n* = 47) and deployment of > 2 FDs (*n* = 9), where isolated evaluation of individual device performance becomes challenging. This yielded 104 evaluable patients for analysis.

Clinical data, aneurysm characteristics, and FU outcomes were retrieved from institutional databases. The observation period ended in March 2025.

Ethics approval was obtained with waived informed consent due to the retrospective nature of the study.

### Imaging and Definitions

All procedures were performed under general anesthesia using a biplane flat-panel system (Azurion, Philips Medical Systems, Amsterdam, Netherlands).

**Fig. 1:**
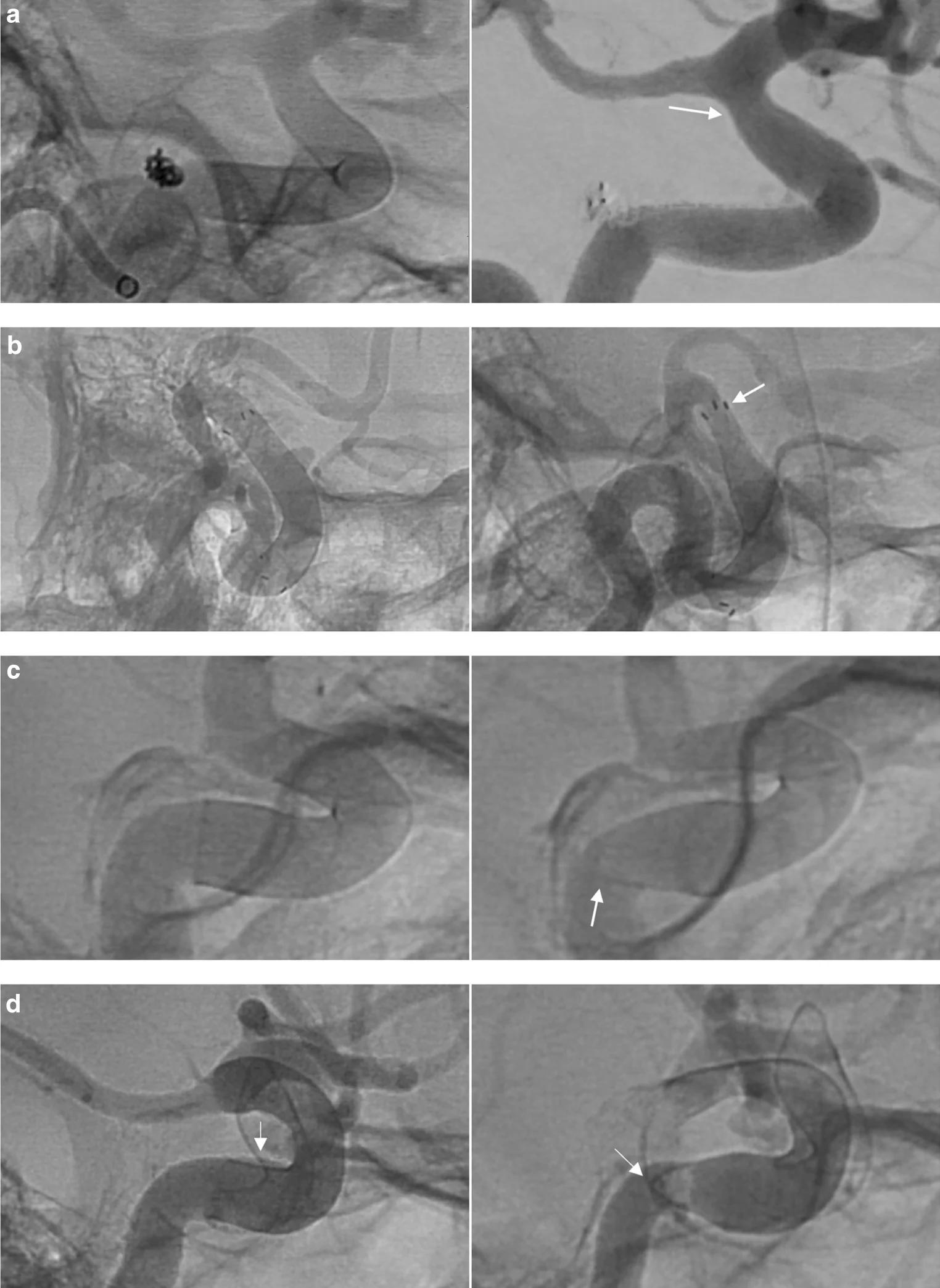
Grading scale for fish-mouthing-like deformities in flow diverter treatment. **a** Pre-fish-mouthing: 45-year-old female patient with a saccular, incidental aneurysm on the RICA. Left: Final run after release of the DED 2 5.0 × 15. Right: In the regular FU after five months, a slight narrowing of the braid can be seen in the distal area of the FD (arrow). **b** Mild fish-mouthing: 30-year-old female patient with two saccular, incidental aneurysms on the RICA. Left: Final run after release of the DED 4.0 × 20. Right: In the regular FU after seven months, the distal part of the FD shows a greater constriction of the braid (arrow). **c** Significant fish-mouthing: 32-year-old female patient with a saccular aneurysm on the LICA. Left: Final run after release of the p64 MW HPC 5.0 × 18. Right: In the regular FU after six months, a prominent fish-mouth-like configuration of the proximal stent end is recognizable (arrow). **d** Severe fish-mouthing: 30-year-old female patient with two saccular, incidental aneurysms on the RICA. Left: Final run after release of the DED 5.0 × 15. In the proximal section, slight dehiscence can be seen between the FD and the vessel wall (arrow). Right: Due to clinical symptoms of hemiparesis, angiographic monitoring is performed after only nine days. This shows the maximum variant of fish-mouthing formation at the proximal end of the stent. Additionally, fish-mouthing-like deformity is observable at the distal end of the affected DED. In the background, a second FD (p64 MW HPC 4.0 × 18) in the LICA demonstrates distal fish-mouthing-like deformity, illustrating multifocal braid deformation in this complex case

Fish-mouthing was defined based on the ESMINT-endorsed Fiehler consensus criteria as focal decrease in the proximal and/or distal stent end compared to the original extent of the device at the respective position [8]. To enhance detection sensitivity and capture the full spectrum of braid deformations, all cases demonstrating device diameter reduction ≥ 10% were classified as fish-mouthing-like deformities. Fish-mouthing severity was classified as pre-fish-mouthing (10–< 25% diameter reduction) and manifest fish-mouthing (≥ 25%), with manifest cases further graded as moderate (25–< 50%), significant (50–< 75%), or severe (≥ 75%). The different degrees of severity are shown in Fig. 1.

Assessment of fish-mouthing-like deformities was performed primarily on unsubtracted, unenhanced digital subtraction angiography (DSA) images obtained at routine FU intervals. In cases where 3D imaging modalities (such as VasoCT) were available, these supplementary datasets were utilized to provide enhanced spatial resolution and confirmation of braid configuration findings.

Two board-certified interventional neuroradiologists (> 10 years FD experience each) independently evaluated all datasets, blinded to angiographic and clinical outcomes. Due to susceptibility artifacts, cases with FU limited to MRI alone were classified as “not assessable”, along with patients who had no available FU imaging.

### Wall Apposition and Procedural Maneuvers

Mechanical optimization of the device-vessel-wall interface was performed at the operator’s discretion. Maneuvers were categorized as:

1. Primary: intra-procedural corrections after FD deployment
2. Secondary: staged corrections in subsequent sessions, and
3. Stent-based and non-stent techniques (e.g. balloon angioplasty, microcatheter, remodeling device).

The frequency of these interventions served as an indirect indicator of wall malapposition. Pre-existing scaffolding stents were excluded.

### Aneurysm Characterization

Aneurysm parameters were systematically extracted from preprocedural data including: morphology, anatomical location, maximum height, width, and neck width (all in mm). Size classification followed established criteria: small (< 10 mm), large (10–< 25 mm), and giant (≥ 25 mm) [13]. For fusiform and dissecting lesions, aneurysm-height-to-distal-vessel-diameter ratios were calculated to assess geometric complexity.

### Device Specifications

FD specifications, including manufacturer, device type, nominal dimensions, DFT construction, and surface modification status, were extracted from procedural records. In cases of telescoping configurations, individual devices were analyzed separately.

### Stent-to-vessel Ratio (SVR)

Parent vessel diameters were measured using threshold-based reconstruction software (Ankyras; Mentice AB, Gothenburg, Sweden), which generates 3D vascular models and calculates diameters from circumferential measurements. The SVR was calculated for proximal (PSVR) and distal (DSVR) positions to determine the degree of FD over- or undersizing using this formula:

$$SVR=\frac{\textit{nominal}\,\textit{stent}\,\textit{diameter}}{\textit{parent}\,\textit{vessel}\,\textit{diameter}}$$

### ICA-tortuosity

The ICA-curvature was assessed using Lin et al.’s method, which measures anterior/posterior genu angles (A, P; °) and inter-genu height difference (d; mm) to derive a tortuosity index defined as d/(A + P). Additionally, curvature was staged into five types (Ia, Ib, II, III, IV), providing qualitative and quantitative metrics [14].

### Outcome Assessment

Aneurysm occlusion was graded using the O’Kelly-Marotta (OKM) grading scale for angiographic imaging and the modified OKM (mOKM) for patients undergoing MRI or CTA [15, 16]. Complete or near-complete occlusion (OKM C–D) was defined as treatment success. All kind of thrombus-associated events were identified through DSA, MRI, or CT imaging. Clinical correlation distinguished radiographic findings from transient or persistent neurological deficits. Device-related neurological outcomes were documented at each FU.

FU imaging followed institutional protocols, typically including DSA at 6 months and MRI or MRA at approximately 24 months, with earlier or additional studies obtained if new clinical symptoms occurred.

Dual antiplatelet therapy (DAPT) adhered to local standards, including periprocedural platelet function testing (Multiplate). Regimens included aspirin combined with clopidogrel, prasugrel, or ticagrelor, with compositions and durations systematically recorded.

### Statistical Analysis

Continuous variables were expressed as means ± standard deviation or medians with interquartile ranges based on Shapiro-Wilk normality testing. Categorical variables were presented as frequencies and percentages.

Between-group comparisons used Student’s t‑test/Mann-Whitney U test for continuous and chi-square/Fisher’s exact test for categorical variables. For multivariable logistic regression analyses, variables were selected based on clinical relevance and expert consensus among the manuscript authors. All candidate variables deemed clinically plausible a priori were included using a forced-entry approach to ensure retention of predetermined clinically important variables regardless of individual statistical significance. Results are presented as adjusted odds ratios (aOR) with 95% CI.

Statistical significance was set at *p* < 0.05 (two-tailed) using SPSS version 29.0.2.0 (IBM Corporation, Armonk, NY, USA).

## Results

### Patient Cohort

A total of 121 FD implantations for the treatment of 127 intracranial aneurysms were analyzed, comprising 116 unruptured and 11 ruptured lesions. Thirty-nine aneurysms were treated with 35 DEDs and 90 aneurysms with 86 FDs of other device-types (p64 Flow Modulation Device, Phenox Wallaby, Bochum, Germany: *n* = 50; p48 Flow Modulation Device, Phenox: *n* = 24; Silk Embolization Device, Balt, Montmorency, France: *n* = 11; PED: *n* = 1). To enhance mechanistic understanding, device-specific subgroup analyses stratified by generation (DED 1 vs. DED 2; p64 1st vs. 2nd generation; Silk 1st vs. 2nd generation) were performed to examine fish-mouthing-like deformities and wall apposition characteristics; detailed findings are provided in Supplemental Material. Baseline characteristics, aneurysm morphology, and antiplatelet regimens were comparable between groups (Table 1). The mean FU duration was 26.0 ± 21.0 months.

**Table 1:** Baseline characteristics

Characteristics	Overall	DED	Non-DED	*P*-value
Number of patients	104	33	76	–
Number of FD	121	35	86	–
Number of aneurysms	127	39	90	–
Number of FD procedures	112	34	80	–
Age (years) (mean ± SD)	55.0 ± 13.4	53.3 ± 14.8	55.2 ± 12.8	0.446
*Sex*	(*n* = 104)	(*n* = 33)	(*n* = 76)	0.603
Female	79 (76.0)	24 (72.7)	59 (77.6)	–
Male	25 (24.0)	9 (27.3)	17 (22.4)	–
*Presentation*	(*n* = 127)	(*n* = 39)	(*n* = 90)	–
Acute SAH	11 (8.7)	2 (5.1)	10 (11.1)	0.144
Asymptomatic	72 (56.7)	26 (66.7)	47 (52.2)	0.159
Recurrence	19 (15.0)	3 (7.7)	17 (18.9)	0.133
Symptoms other than SAH	25 (19.7)	8 (20.5)	16 (17.8)	0.908
*Circulation*	(*n* = 127)	(*n* = 39)	(*n* = 90)	0.240
Anterior	100 (78.7)	33 (84.6)	68 (75.6)	–
Posterior	27 (21.3)	6 (15.4)	22 (24.2)	–
*Location*	(*n* = 127)	(*n* = 39)	(*n* = 90)	–
ICA	79 (62.2)	29 (74.4)	50 (55.6)	0.051
Basilar artery	9 (7.1)	5 (12.8)	4 (4.4)	0.128
MCA	3 (2.4)	1 (2.6)	2 (2.2)	1.000
PCA	8 (6.3)	0 (0.0)	8 (8.9)	0.105
ACA	4 (3.1)	1 (2.6)	3 (3.3)	1.000
ACoA	9 (7.1)	1 (2.6)	9 (10.0)	0.281
PCoA	4 (3.1)	1 (2.6)	4 (4.4)	1.000
Vertebral artery	7 (5.5)	1 (2.6)	6 (6.7)	0.674
SCA	1 (0.8)	0 (0.0)	1 (1.1)	1.000
AICA	1 (0.8)	0 (0.0)	1 (1.1)	1.000
PICA	2 (1.6)	0 (0.0)	2 (2.2)	1.000
*Morphology*	(*n* = 127)	(*n* = 39)	(*n* = 90)	–
Saccular	99 (78.0)	30 (76.9)	70 (77.8)	1.000
Fusiform	18 (14.2)	8 (20.5)	10 (11.1)	0.069
Blister-like	4 (3.1)	1 (2.6)	4 (4.4)	1.000
Dissecting	5 (3.9)	0 (0.0)	5 (5.6)	0.117
Mycotic	1 (0.8)	0 (0.0)	1 (1.1)	1.000
*Size*	(*n* = 127)	(*n* = 39)	(*n* = 90)	*–*
Small (< 10 mm)	100 (78.7)	23 (59.0)	79 (87.8)	< 0.001
Large (10–< 25 mm)	25 (19.7)	15 (38.5)	10 (11.1)	< 0.001
Giant (≥ 25 mm)	2 (1.6)	1 (2.6)	1 (1.1)	0.515
Neck width (mm) (mean ± SD)	5.85 ± 6.43	8.65 ± 9.86	4.58 ± 3.51	0.004
Ratio aneurysm height/distal vessel diameter (mean ± SD)	3.01 ± 1.78	2.89 ± 1.59	3.08 ± 1.92	1.000
*Surface modification*	(*n* = 121)	(*n* = 35)	(*n* = 86)	0.001
Yes	48 (39.7)	6 (17.1)	42 (48.8)	–
No	73 (60.3)	29 (82.9)	44 (51.2)	–
*DFT technology*	(*n* = 121)	(*n* = 35)	(*n* = 86)	0.001
Yes	100 (82.6)	35 (100.0)	65 (75.6)	–
No	21 (17.4)	0 (0.0)	21 (24.4)	–
*FD diameter*	(*n* = 121)	(*n* = 35)	(*n* = 86)	–
Nominal diameter (mm) (median [IQR])	4.0 (3.0–4.5)	4.5 (4.0–5.0)	4.0 (3.0–4.0)	< 0.001
PSVR (mean ± SD)	1.01 ± 0.21	1.09 ± 0.18	0.98 ± 0.21	0.002
DSVR (mean ± SD)	1.19 ± 0.26	1.22 ± 0.21	1.18 ± 0.28	0.161
*Occlusion rate (OKM, mOKM)*				
Time period 1 (3–9 mo.)	89 (79.5)	26 (76.5)	63 (80.8)	0.605
Time period 2 (up to 24 mo.)	107 (84.9)	32 (84.2)	75 (85.2)	0.884
Time period 3 (final FU)	110 (87.3)	32 (84.2)	78 (88.6)	0.563
Mean FU-time (months) (mean ± SD)	26.0 ± 21.0	27.3 ± 22.1	25.4 ± 20.6	0.754
*DAPT*				
Length of DAPT (months) (mean ± SD)	8.1 ± 3.6	8.7 ± 4.5	7.8 ± 3.0	0.303

All data are presented as *n* (%) unless specified otherwise.

*DED* DERIVO Embolization Device; *FD* Flow diverter; *SAH* subarachnoid hemorrhage; *ICA* internal carotid artery; *MCA* middle cerebral artery; *PCA* posterior cerebral artery; *ACA* anterior cerebral artery; *ACoA* anterior communicating artery; *PCoA* posterior communicating artery; *SCA* superior cerebellar artery; *AICA* anterior inferior cerebellar Artery; *PICA* posterior inferior cerebellar Artery; *DFT* drawn filled tubing; *PSVR* proximal stent-to-vessel ratio; *DSVR* distal stent-to-vessel ratio; *OKM* O’Kelly Marotta grading scale; *mOKM* modified O’Kelly Marotta grading scale; *FU* Follow Up; *DAPT* dual antiplatelet therapy.

### Multivariable Analysis for Baseline Imbalances

A multivariable logistic regression (*n* = 121) showed good discrimination (79.3% accuracy) and calibration (Nagelkerke R^2^ = 0.338; Hosmer-Lemeshow *p* = 0.796). Large aneurysm size (≥ 10 mm) was the strongest independent predictor of DED use (aOR 6.95; *p* = 0.002). Younger age also favored DED selection (per year, aOR 0.95; *p* = 0.016), and neck width showed a trend toward significance (per mm, aOR 1.09; *p* = 0.056). Neither anterior circulation (aOR 0.46; *p* = 0.234), fusiform morphology (aOR 1.16; *p* = 0.849), nor aSAH presentation (aOR 0.32; *p* = 0.112) were independent predictors.

### Fish-mouthing-like Deformities

Of 121 deployed devices, 113 (93.4%) were evaluable for fish-mouthing assessment. Fish-mouthing-like deformities occurred significantly more often in the DED group (16/32, 50.0%) than in the non-DED group (22/81, 27.2%; *p* = 0.021). This difference was primarily attributable to pre-fish-mouthing (10/32, 31.3% vs. 12/81, 14.8%; *p* = 0.047), whereas manifest deformities (≥ 25% diameter reduction) showed no significant difference (6/32, 18.8% vs. 10/81, 12.3%; *p* = 0.382). Overall, the anatomical distribution demonstrated distal predominance (26/38, 68.4% vs. 17/38, 44.7% proximal), while proximal deformities were significantly more frequent with DED (9/16, 56.3% vs. 8/22, 36.4%; *p* = 0.014). Intraprocedural fish-mouthing-like deformity was rare and showed no intergroup difference (2/16, 12.5% vs. 2/22, 9.1%; *p* = 0.640). Importantly, age-stratified analysis revealed that younger age was associated with a higher likelihood of fish-mouthing-like deformities in both DED (*p* = 0.015) and non-DED (*p* = 0.031). This age effect was mainly attributable to manifest deformities, which demonstrated pronounced age-dependent prevalence in both DED (*p* < 0.001) and non-DED (*p* = 0.043) groups, whereas pre-fish-mouthing showed no age association (DED: *p* = 0.826; non-DED: *p* = 0.582). No association was found between fish-mouthing severity and aneurysm size, location, or ICA-tortuosity. Neither DFT technology nor surface modification showed significant associations with fish-mouthing-like deformities in the overall cohort (DFT: *p* = 0.291; surface modification: *p* = 0.850) or in device subgroups (*p* > 0.05).

### Wall Apposition Maneuvers

Overall mechanical wall apposition optimization was required more frequently in the DED cohort (21/35, 60.0%) than in other FD-types (22/86, 25.6%; *p* < 0.001). Both stent-based (10/35, 28.6% vs. 6/86, 7.0%; *p* = 0.001) and non-stent techniques (16/35, 45.7% vs. 19/86, 22.1%; *p* = 0.009) contributed to this difference. This disparity was mainly attributable to primary, stent (7/35, 20.0% vs. 3/86, 3.5%; *p* = 0.003) and non-stent (16/35, 45.7% vs. 17/86, 19.8%; *p* = 0.004) maneuvers during the index procedure (18/35, 51.4% vs. 20/86, 23.3%; *p* = 0.002), as secondary interventions were comparable between subgroups (4/35, 11.4% vs. 4/86, 4.7%; *p* = 0.174). Balloon angioplasty was the most common primary maneuver with higher rates in DED patients (14/35, 40.0% vs. 12/86, 14.0%; *p* = 0.003).

Proximal malapposition during the index procedure represented the most common indication for intervention. Specifically, proximal stent-based corrections were needed in 17% (6/35) of DEDs versus 2% (2/86) of other FDs (*p* = 0.003), while non-stent-based maneuvers such as balloon angioplasty or microcatheter reshaping were performed proximally in 43% (15/35) versus 17% (15/86), respectively (*p* = 0.003).

**Fig. 2:**
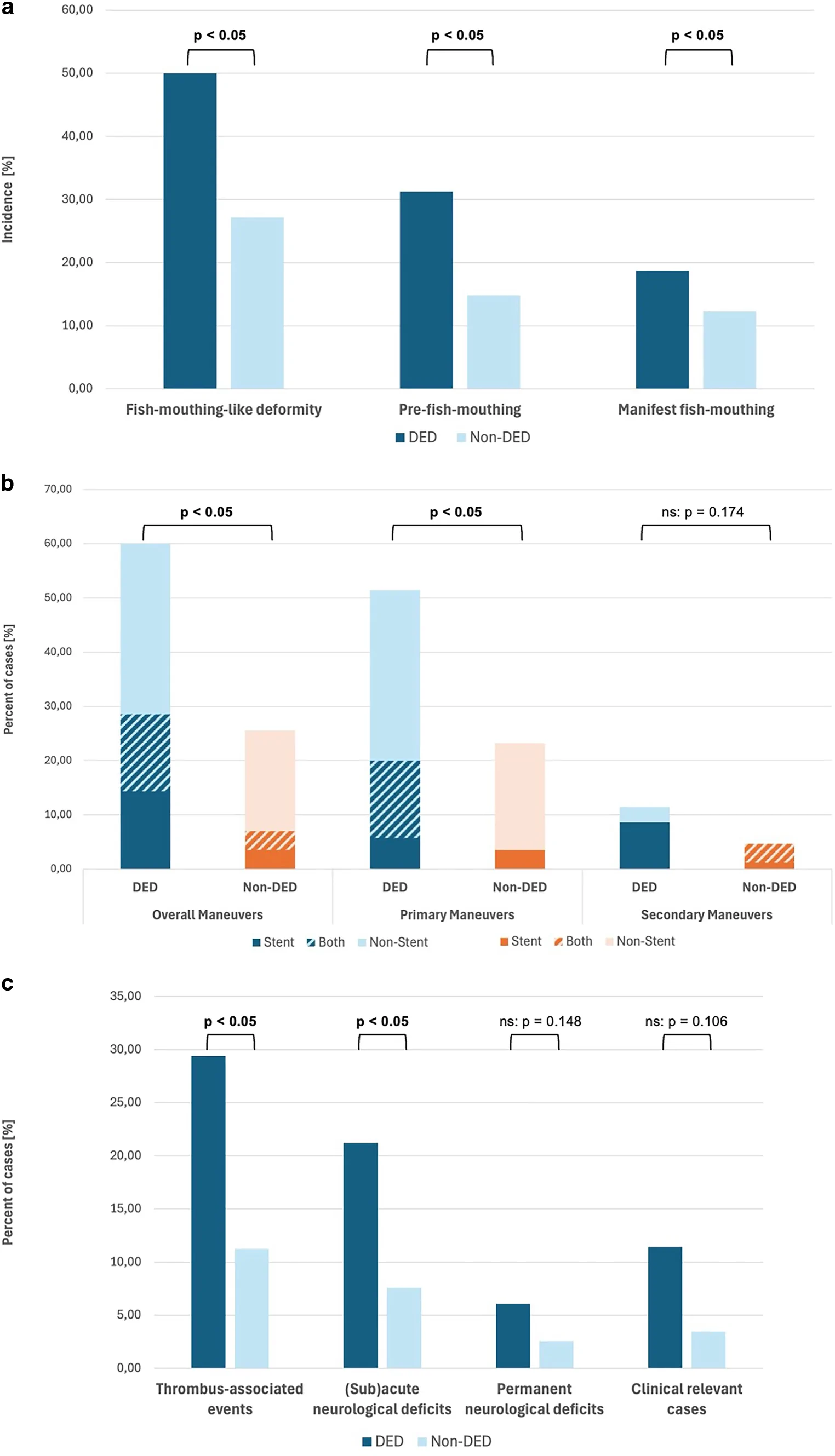
Comprehensive results overview. **a** Representation of the incidence of fish-mouthing-like deformities by device type. **b** Representation of the distribution of mechanical wall apposition maneuvers by device type and strategy. **c** Representation of the clinically relevant outcomes by device type. Clinically relevant outcomes were defined as cases requiring retreatment and/or demonstrating permanent neurological deficit (DED: 4/35, 11.4% vs. non-DED: 3/86, 3.5%; *p* = 0.106). *DED* DERIVO Embolization Device

More detailed results on fish-mouthing-like deformities and wall apposition maneuvers are presented in Table 2 and Fig. 2.

**Table 2:** Fish-mouthing-like deformities and wall apposition interventions—device-specific comparison

Parameter	DED (*n* = 32–35)	Non-DED (*n* = 81–86)	*P*-value/OR (95%–CI)
***Fish-mouthing-like deformities***			
**Assessable cases**	32/35 (91.4%)	81/86 (94.2%)	0.689/0.66 (0.15–2.92)
**Incidence of fish-mouthing-like deformities**	16/32 (50.0%)	22/81 (27.2%)	0.021/2.68 (1.15–6.23)
**Incidence of pre-fish-mouthing**	10/32 (31.3%)	12/81 (14.8%)	0.047/2.61 (0.99–6.87)
**Incidence of manifest fish-mouthing**	6/32 (18.8%)	10/81 (12.3%)	0.382/1.64 (0.54–4.96)
**Severity**			
Moderate	3/32 (9.4%)	7/81 (8.6%)	0.703/1.09 (0.26–4.52)
Significant	1/32 (3.1%)	3/81 (3.7%)	1.000/0.84 (0.08–8.37)
Severe	2/32 (6.3%)	0/81 (0.0%)	0.078/13.36 (0.62–286.26)
**Anatomical distribution of fish-mouthing-like deformities**			
Proximal	9/32 (28.1%)	8/81 (9.9%)	0.021/2.68 (1.15–6.23)
Distal	10/32 (31.3%)	16/81 (19.8%)	0.219/1.85 (0.73–4.66)
**Retreatment of fish-mouthing cases**	2/16 (12.5%)	1/22 (4.5%)	0.562/3.00 (0.25–36.33)
**Time to detection (months) (mean** **±** **SD)**	5.0 ± 2.8	5.6 ± 2.5	0.531
***Wall apposition maneuvers***			
**Overall maneuvers**	21/35 (60.0%)	22/86 (25.6%)	< 0.001/4.36 (1.90–10.03)
Stent-assisted technique	10/35 (28.6%)	6/86 (7.0%)	0.001/5.33 (1.76–16.14)
Non-stent technique	16/35 (45.7%)	19/86 (22.1%)	0.009/2.97 (1.23–6.86)
**Primary maneuvers**	18/35 (51.4%)	20/86 (23.3%)	0.002/3.49 (1.52–8.02)
*Stent-assisted technique*	7/35 (20.0%)	3/86 (3.5%)	0.003/6.92 (1.67–28.58)
*Non-stent technique*	16/35 (45.7%)	17/86 (19.8%)	0.004/3.42 (1.46–8.00)
*Anatomical distribution of primary maneuvers*			
Proximal stent	6/35 (17.1%)	2/86 (2.3%)	0.003/8.96 (1.66–45.48)
Proximal non-stent	15/35 (42.9%)	15/86 (17.4%)	0.003/3.55 (1.49–8.48)
Distal stent	1/35 (2.9%)	2/86 (2.3%)	1.000/1.24 (0.11–14.06)
Distal non-stent	7/35 (20.0%)	11/86 (12.8%)	0.354/1.71 (0.60–4.84)
**Secondary maneuvers**	4/35 (11.4%)	4/86 (4.7%)	0.174/2.65 (0.62–11.23)

All data are presented as *n* (%) unless specified otherwise.

The sum of proximal and distal fish-mouthing-like deformity frequencies exceeds the total number of fish-mouthing-like deformities, as some devices exhibited fish-mouthing-like deformities at both the proximal and distal device ends simultaneously.

The reported overall number of mechanical wall apposition optimization maneuvers refers to the number of FDs requiring at least one mechanical intervention. In some cases, both stent-based and non-stent-based procedures were used, so that the sum of the individual maneuvers exceeds the total number of cases that required a maneuver.

*DED* DERIVO Embolization Device; *Non-Stent* Balloon angioplasty, microcatheter, distal access catheter, or remodeling device utilization

### Multivariable Predictive Models

#### Independent Predictors of Fish-mouthing-like Deformities

A multivariable logistic regression (*n* = 113) achieved significance (Omnibus χ^2^ = 20.8, df = 5; *p* = 0.001) with excellent calibration (Nagelkerke R^2^ = 0.233; Hosmer-Lemeshow *p* = 0.937; 72.6% accuracy). DED implantation independently predicted fish-mouthing (aOR 3.00; *p* = 0.030), as did younger patient age (aOR 0.94 per year; *p* = 0.004). Aneurysm size (aOR 1.04; *p* = 0.474), neck width (aOR 0.89; *p* = 0.182), and gender (aOR 0.93; *p* = 0.887) were not significant.

#### Independent Predictors of Wall Apposition Interventions

A multivariable logistic regression (*n* = 121) was non-significant overall (Omnibus χ^2^ = 8.69, df = 5; *p* = 0.122) with modest variance explanation (Nagelkerke R^2^ = 0.127) but good calibration (Hosmer-Lemeshow *p* = 0.770; 64.4% accuracy). Only DED implantation independently predicted adjunctive interventions (aOR 3.47; *p* = 0.013). Aneurysm size (aOR 1.06; *p* = 0.214), neck width (aOR 0.96; *p* = 0.547), circulation territory (aOR 0.06; *p* = 0.319), tortuosity index (aOR 1.23; *p* = 0.865), and PSVR (aOR 0.90; *p* = 0.593) were not significant predictors.

#### Summary of Predictors

DED use was the only consistent independent predictor of (1) the occurrence of fish-mouthing-like deformities and (2) the need for wall apposition maneuvers. Aneurysm size, neck width, location, FD specifications, PSVR, and vessel tortuosity showed no significant influence.

### Aneurysm Occlusion

All 104 patients were considered for analysis, with 2 patients (1.9%) lost to FU, yielding outcome data for 119 of 121 implanted FD. Final aneurysm occlusion assessment encompassed 127 treated aneurysms; occlusion rates were calculated per treated aneurysm by dividing the number of aneurysms achieving complete or near-complete occlusion (OKM C–D) by the total number of evaluable aneurysms at each FU timepoint. At final angiographic or cross-sectional FU, complete or near-complete occlusion (OKM C–D) was achieved in 84.2% of DED-treated and 88.6% of non-DED aneurysms (*p* = 0.563). Occlusion rates increased over time in both groups without significant intergroup differences at any interval (see Table 1).

### Thromboembolic and Neurological Outcomes

Periprocedural or delayed thrombus-associated events occurred more frequently in the DED cohort (10/34, 29.4%) compared with other FD-types (9/80, 11.3%; *p* = 0.008). Same applies to transient neurological deficits, these affected more DED-cases (7/33, 21.2% vs. 6/79, 7.6%; *p* = 0.010). Most thrombus-associated events were clinically silent or transient, detected as DWI-positive lesions without permanent neurological deficit (8/10, 80.0% vs. 7/9, 77.8%; *p* = 1.000). Persistent neurological morbidity attributable to the device was low and comparable between groups (2/33, 6.0% vs. 2/78, 2.6%, *p* = 0.148). No procedure-related deaths occurred.

### SVR-correlations

In the overall cohort, no significant association was found between stent-to-vessel ratios and fish-mouthing-like deformities (DSVR vs. distal deformity: r = 0.099, *p* = 0.120; PSVR vs. proximal deformity: r = −0.011, *p* = 0.987). The same holds true for the non-DED subgroup (distal deformity: r = −0.001, *p* = 0.599; proximal deformity: r = 0.017, *p* = 0.694). Only in the DED subgroup there is a nearly significant correlation between DSVR and distal deformity (r = 0.402, *p* = 0.062), but not between PSVR and proximal deformity (r = −0.252, *p* = 0.104).

## Discussion

### Principal Findings

In this single-center analysis DEDs exhibited higher rates of fish-mouthing-like deformities—particularly pre-fish-mouthing—and wall malapposition than other FDs. Manifest fish-mouthing (≥ 25% diameter reduction) was observed at similar rates across FD-types, yet younger age independently predicted its occurrence, suggesting that increased vascular reactivity or enhanced endothelial remodeling in younger patients may contribute beyond device-specific mechanical factors. DED deployment required more frequent primary, intraprocedural wall apposition maneuvers, particularly proximally, yet long-term aneurysm occlusion, DAPT duration, and permanent neurological outcomes were comparable between device groups. Thrombus-associated events were more frequent with DEDs but were predominantly silent or transient.

### Interpretation and Potential Mechanisms

Fish-mouthing-like deformities and wall apposition represent distinct phenomena. Fish-mouthing is typically a delayed structural braid deformation, whereas wall apposition reflects the immediate device-vessel interface after implantation. In this study, both parameters were evaluated together to explore device-specific deployment behavior in a hypothesis-generating manner, without implying a causal relationship.

Minor caliber changes at the device ends—such as pre-fish-mouthing—may, in part, reflect physiological stent integration and remodeling rather than unequivocal pathology and may lack immediate clinical significance. Nevertheless, the clear differences between FD-types regarding these changes may represent enhanced healing responses that could predispose to more clinically relevant changes during the integration period. The device-specific predominance of proximal irregularities in the DED cohort points to apposition dynamics at the landing zone as a plausible contributor. Two, not mutually exclusive, mechanisms merit consideration.

Inadequate-wall-apposition hypothesis. If local radial support is insufficient at the landing zone, blood can track between the device and vessel wall; pulsatile forces may then urge struts inward, yielding a tapered (“fish-mouth”) end. This mechanism would preferentially affect the proximal ICA segment (transition from petrous to cavernous), where curvature and changes in compliance are greatest [17].

Oversizing-related effects. Elevated SVRs may generate excessive radial force triggering constrictive vessel responses from endothelial and vascular smooth muscle cells, resulting in concomitant narrowing of both the vessel and the FD mesh. Distally, where parent vessel caliber often physiologically decreases and SVRs increases, conventional oversizing effects may still operate [9]. Our observation of a near-significant correlation between distal SVR and distal fish-mouthing-like deformations in the DED subgroup is compatible with this mechanism.

Importantly, mechanical design characteristics beyond a single material choice likely modulate in-vivo braid behavior—e.g., wire stiffness, braid angle, and alloy/surface properties (including but not limited to DFT constructions). Thus, while design intent (deliverability, hemocompatibility, apposition) has advanced, inter-platform mechanical variability can translate into distinct deformation patterns that warrant device-specific evaluation. DFT constructions serves as a prime example of this concept: laboratory investigations have demonstrated that DFT technology, wherein metallic-wires are drawn over an inner platinum core, results in reduced wire stiffness compared to conventional solid-wire constructions when bent sharply [18]. This mechanical alteration may compromise the radial force generation necessary for optimal wall apposition, particularly in challenging anatomical configurations and may lead to fish-mouthing-like deformities [10].

### Comparative Device Performance

Reported rates of fish-mouthing vary widely due to heterogeneous definitions and imaging protocols [4, 8]. When comparisons are restricted to manifest deformities—where thresholds are more comparable—our incidence with DEDs lies toward the upper end of published ranges. This likely reflects (i) our systematic, sensitive detection of braid changes and (ii) inconsistent reporting standards across studies.

The substantial heterogeneity in reported DED fish-mouthing rates across contemporary literature—ranging from 1.1% in the multicenter BRAIDED study [19], through 4.2% in Goertz et al.’s series [20], to 11.5% in Taschner et al.’s series [2]—reflects these methodological inconsistencies that compromise inter-study comparability. The Indian REMIND study’s exceptional 1% fish-mouthing rate [21] and Kaschner et al.’s 0% rate [22] may reflect population-specific factors, different anatomical locations, or institutional techniques rather than fundamental device performance differences.

Recent series of second-generation DEDs describe lower deformation rates, supporting the notion that iterative design updates may mitigate some phenomena observed with earlier generations: Schwab et al. [23] documented 4.8% with DED 2 Heal devices, while Thormann et al. [24] observed 2.7% with the DED 2.

We stratified DED into first- and second-generation groups and observed no crucial significant differences; detailed results are provided in the Supplementary.

Nevertheless, direct cross-study comparisons should be made cautiously until standardized criteria for detection and grading are consistently applied.

### Wall Apposition Enhancement

The significant increase in requirement for mechanical wall apposition optimization—particularly at the proximal device end and during the index procedure—with DED represents a substantial heightening in procedural complexity compared to alternative FD-types, suggesting that DED deployment requires enhanced operator expertise and procedural planning. Our institutional approach emphasizes achieving near-perfect wall apposition, correcting even minor device-vessel interface irregularities—a strategy that may amplify intervention rates compared with centers employing less stringent thresholds.

Balloon remodeling and additional stenting emerged as the most frequently employed techniques at our center with comparable rates across studies. Wall apposition enhancement strategies have varied considerably across published series, with balloon angioplasty rates ranging from 5.2% to 34.6% in first-generation DED cohorts. Akgul et al. [25] observed adjunctive use of balloon angioplasty in 34.6% of cases, while Trivelato et al. [19] and Taschner et al. [2] reported rates of 20.5% and 18.8%, respectively. Lower frequencies were noted by Goertz et al. [26] (5.8%) and Mahajan et al. [21] (5.2%), whereas Pinana et al. [27] documented intervention in 16.4%. Second-generation DED studies report lower rates ranging from 6% to 10% as described by Schwab et al. [23] and Goertz et al. [28].

Intraprocedural stenting for proximal malapposition was required in 27.8% of large-diameter DED cases in Butt et al.’s cohort [29], whereas in Goertz et al.’s series 12% of cases necessitated either stenting or balloon angioplasty [20].

### Clinical Implications

From a practical standpoint, operators should anticipate a higher probability of intraprocedural wall apposition optimization when using DEDs, especially proximally. This might have implications for procedural planning (balloon availability, adjunctive stenting strategy, catheter support) and patient counseling (potential need for corrective maneuvers during index procedure). Of note, almost all DEDs used in our cohort (all but one case) corresponded to configurations with distally located flared ends. Therefore, the potential influence of flare-end position on the observed proximal-distal distribution of fish-mouthing-like deformities cannot be reliably assessed in this dataset. Crucially, in our cohort these technical demands did not translate into inferior long-term aneurysm occlusion or worse permanent neurological outcomes. The observed increase in thrombus-associated events—largely silent or transient—emphasizes the importance of rigorous antiplatelet management, careful device sizing, and high-quality intraprocedural imaging to optimize wall apposition.

### Limitations

As a retrospective, single-center study, our findings are subject to potential selection bias and limited generalizability, necessitating that the results should be interpreted with caution. The FD under discussion represents an effective and safe device for the treatment of intracranial aneurysms and, in our view, continues to hold excellent indications. A critical confounder is the selection bias toward more complex aneurysms in the DED cohort. The DED represents a valuable treatment option for such cases and was preferentially used for inherently higher-risk pathologies, including a disproportionately higher, though not statistically significant, number of basilar artery and fusiform aneurysms. This bias, driven by the availability of DEDs in larger diameters and greater lengths, likely contributes to the higher clinical event rate observed.

Although our single-center findings suggest that this FD may be somewhat more challenging to implant and might require additional use of devices, these observations should be verified within the framework of a multicenter study, ideally embedded within a prospective trial. Under no circumstances should premature conclusions be drawn regarding an increased rate of actual complications.

Additionally, our readings were very sensitive regarding minor insufficiencies in wall apposition and fish-mouthing exceeding clinically relevant events.

## Conclusion

DEDs were associated with more frequent wall apposition optimization, particularly during the index procedure and at the proximal device end, and higher rates of pre-fish-mouthing, while manifest fish-mouthing, aneurysm occlusion, and permanent neurological outcomes were comparable to other FDs. These findings are hypothesis-generating and support careful procedural planning with DEDs, while underscoring the need for larger, preferably prospective multicenter studies before drawing definitive conclusions.

## Supplementary Information


Supplemental Material Clean Version


## Data Availability

Study data is available from the corresponding author upon reasonable request by a qualified investigator, and after clearance by the local institutional review board.

## References

[1] Gaub M, Murtha G, Lafuente M, Webb M, Luo A, Birnbaum LA, et al. Flow Diversion for Endovascular Treatment of Intracranial Aneurysms: Past, Present, and Future Directions. J Clin Med. 2024; 10.3390/jcm13144167. 39064207 PMC11278297

[2] Taschner CA, Stracke CP, Dorn F, Kadziolka KB, Kreiser K, Solymosi L, et al. Derivo embolization device in the treatment of unruptured intracranial aneurysms: a prospective multicenter study. J Neurointerv Surg. 2021;13(6):541–6. 10.1136/neurintsurg-2020-016303. 32900908 PMC8142444

[3] Zhang Q, Shao Q, Chang K, Sheng Z, Zhang H, Chen Y, et al. A Proposed Grading Scale Based on Radiographic Imaging for Wall Apposition to Estimate Neurological Complications after Flow Diverter Treatment. AJNR Am J Neuroradiol. 2025; 10.3174/ajnr.A8785. 40210458 PMC12633668

[4] Ortega-Gutierrez S, Rodriguez-Calienes A, Vivanco-Suarez J, Cekirge HS, Hanel RA, Dibas M, et al. Braid stability after flow diverter treatment of intracranial aneurysms: a systematic review and meta-analysis. J Neurointerv Surg. 2023; 10.1136/jnis-2023-021120. 38124177

[5] Torche E, Cao R, Mattar A, Laubacher M, Riva R, Eker OF. Ultra-Early “Fishmouth stenosis” and thrombosis of a Surpass Evolve flow diversion device following treatment of multiples right siphon aneurysms. Neuroradiology. 2023;65(12):1803–7. 10.1007/s00234-023-03239-1. 37845483

[6] Kocer N, Mondel PK, Yamac E, Kavak A, Kizilkilic O, Islak C. Is there an association between flow diverter fish mouthing and delayed-type hypersensitivity to metals?-a case-control study. Neuroradiology. 2017;59(11):1171–8. 10.1007/s00234-017-1910-3. 28875355

[7] Rodriguez-Erazu F, Cortez GM, Lopes DK, Gutierrez-Aguirre SF, De Toledo OF, Aghaebrahim A, et al. Braids and beyond: a comprehensive study on pipeline device braid stability from PREMIER data. J Neurointerv Surg. 2025; 10.1136/jnis-2024-022350. 39357888

[8] Fiehler J, Ortega-Gutierrez S, Anagnostakou V, Cortese J, Cekirge HS, Fiorella D, et al. Evaluation of flow diverters for cerebral aneurysm therapy: recommendations for imaging analyses in clinical studies, endorsed by ESMINT, ESNR, OCIN, SILAN, SNIS, and WFITN. J Neurointerv Surg. 2025;17(6):632–9. 10.1136/jnis-2023-021404. 38830670 PMC12171491

[9] Vladev G, Sirakov A, Matanov S, Sirakova K, Ninov K, Sirakov S. Subacute Stent Deformities as an Underlying Reason for Vessel Stenosis after Flow Diversion with the p64 Stent: Review and Discussion of Biologic Mechanisms and Consequences. AJNR Am J Neuroradiol. 2025;46(4):712–9. 10.3174/ajnr.A8564. 40113252 PMC11979843

[10] Popica DA, Cortese J, Oliver AA, Plaforet V, Molina Diaz I, Rodriguez-Erazu F, et al. Flow diverter braid deformation following treatment of cerebral aneurysms: incidence, clinical relevance, and potential risk factors. J Neurointerv Surg. 2025; 10.1136/jnis-2024-022236. 39214688

[11] Maragkos GA, Dmytriw AA, Salem MM, Tutino VM, Meng H, Cognard C, et al. Overview of Different Flow Diverters and Flow Dynamics. Neurosurgery. 2020;86(Suppl 1):S21–S34. 10.1093/neuros/nyz323. 31838536

[12] von Elm E, Altman DG, Egger M, Pocock SJ, Gotzsche PC, Vandenbroucke JP, et al. The Strengthening the Reporting of Observational Studies in Epidemiology (STROBE) statement: guidelines for reporting observational studies. Lancet. 2007;370(9596):1453–7. 10.1016/S0140-6736(07)61602-X. 18064739

[13] Lylyk I, Scrivano E, Lundquist J, Ferrario A, Bleise C, Perez N, et al. Pipeline Embolization Devices for the Treatment of Intracranial Aneurysms, Single-Center Registry: Long-Term Angiographic and Clinical Outcomes from 1000 Aneurysms. Neurosurgery. 2021;89(3):443–9. 10.1093/neuros/nyab183. 34098575 PMC8374967

[14] Lin LM, Colby GP, Jiang B, Uwandu C, Huang J, Tamargo RJ, et al. Classification of cavernous internal carotid artery tortuosity: a predictor of procedural complexity in Pipeline embolization. J Neurointerv Surg. 2015;7(9):628–33. 10.1136/neurintsurg-2014-011298. 24996435

[15] O’Kelly CJ, Krings T, Fiorella D, Marotta TR. A novel grading scale for the angiographic assessment of intracranial aneurysms treated using flow diverting stents. Interv Neuroradiol. 2010;16(2):133–7. 10.1177/159101991001600204. 20642887 PMC3277972

[16] Miyazaki A, Nishio M, Fujita A, Kohta M, Kojita Y, Horii S, et al. Predicting the O’Kelly-Marotta scale score after flow-diverter stent placement using silent MRA. Jpn J Radiol. 2024;42(12):1403–12. 10.1007/s11604-024-01632-1. 39207642 PMC11588759

[17] Gao H, You W, Wei D, Lv J, Sun W, Li Y. Tortuosity of parent artery predicts in-stent stenosis after pipeline flow-diverter stenting for internal carotid artery aneurysms. Front Neurol. 2022;13:1034402. 10.3389/fneur.2022.1034402. 36313497 PMC9596983

[18] Schaffer JE, Gordon R. Engineering Characteristics of Drawn Filled Nitinol Tube. In: Pelton AR, Duerig T, editors. International Conference on Shape Memory and Superelastic Technologies. Pacific Grove, California, USA: SMST Society, Inc.; 2004. pp. 1–9.

[19] Trivelato FP, Abud DG, Ulhoa AC, Waihrich ES, Abud TG, Castro Afonso LH, et al. Derivo Embolization Device for the Treatment of Intracranial Aneurysms. Stroke. 2019;50(9):2351–8. 10.1161/STROKEAHA.119.025407. 31288675

[20] Goertz L, Zopfs D, Kottlors J, Pennig L, Schob S, Schlamann M, et al. Treatment of intracranial aneurysms with large-diameter (〉/=5.5 mm) Derivo Embolization Devices, with particular focus on 7 and 8 mm diameter devices. Interv Neuroradiol. 2024;15910199241248479. 10.1177/15910199241248479. PMC11571303 38706147

[21] Mahajan NP, Mushtaq M, Bhatti A, Purkayastha S, Dange N, Cherian M, et al. REtrospective Multicenter INdian Study of Derivo Embolization Device (REMIND): Periprocedural Safety. Neurointervention. 2021;16(3):232–9. 10.5469/neuroint.2021.00227. 34425637 PMC8561030

[22] Kaschner MG, Petridis A, Turowski B. Single-center experience with the new generation Derivo Embolization Device in ruptured dissecting and blister aneurysms. Acta Radiol. 2020;61(1):37–46. 10.1177/0284185119852731. 31166695

[23] Schwab R, Kabbasch C, Goertz L, Kaschner M, Weiss D, Loehr C, et al. The DERIVO 2 Heal Embolization Device in the Treatment of Ruptured and Unruptured Intracranial Aneurysms: a Retrospective Multicenter Study. Clin Neuroradiol. 2025;35(1):25–34. 10.1007/s00062-024-01446-8. 39172220 PMC11832578

[24] Thormann M, Sillis N, Thoma T, Altenbernd J, Berger B, Cioltan A, et al. The DERIVO(R)2 Embolization Device in the treatment of ruptured and unruptured intracranial aneurysms: A multicenter analysis. Interv Neuroradiol. 2024;30(5):672–8. 10.1177/15910199221142643. 36567499 PMC11569461

[25] Akgul E, Onan HB, Akpinar S, Balli HT, Aksungur EH. The DERIVO Embolization Device in the Treatment of Intracranial Aneurysms: Short- and Midterm Results. World Neurosurg. 2016;95:229–40. 10.1016/j.wneu.2016.07.101. 27514698

[26] Goertz L, Zopfs D, Kottlors J, Borggrefe J, Pennig L, Schlamann M, et al. Long-term Safety and Efficacy of the Derivo Embolization Device in a Single-center Series. Clin Neuroradiol. 2024;34(4):789–98. 10.1007/s00062-024-01423-1. 38814452 PMC11564379

[27] Pinana C, Remollo S, Zamarro J, Werner M, Espinosa de Rueda M, Vega P, et al. Derivo embolization device for intracranial aneurysms: a Spanish multicenter retrospective study. J Neurointerv Surg. 2023;15(9):871–5. 10.1136/jnis-2022-019220. 35999049

[28] Goertz L, Zopfs D, Schonfeld M, Zaeske C, Pennig L, Brinker G, et al. First clinical experience with the Derivo 2heal embolization device for the treatment of intracranial aneurysms. Interv Neuroradiol. 2023:15910199231193577. 10.1177/15910199231193577. PMC12852631 37574801

[29] Butt W, Kim CN, Ramaswamy R, Smith A, Maliakal P. Implantation of Large Diameter (5.5–6 mm) Derivo Embolization Devices for the Treatment of Cerebral Aneurysms. Clin Neuroradiol. 2022;32(2):481–9. https://doi.org/10.1007/s00062-021-01086‑2. 10.1007/s00062-021-01086-2 34498094

