# Supplementary material for: Incidence of Fish-mouthing and Wall Malapposition of Different Flow Diverters in the Treatment of Cerebral Aneurysms: Device-specific Findings in a Retrospective Single-center Analysis
Source: Clin Neuroradiol. 2026 Apr 15;36(3):963–75. doi: 10.1007/s00062-026-01651-7 (PMC13522061; doi:10.1007/s00062-026-01651-7)
Supplement: Supplementary file 1 [file 62_2026_1651_MOESM1_ESM.docx]

**Supplemental Material**

**Title: Incidence of fish-mouthing and wall malapposition of different flow diverters in the treatment of cerebral aneurysms: Device-specific findings in a retrospective single-center analysis**

**Authors**: Maximilian Rothe^1^, Martin Renz^1^, Maria Berndt-Mück^1^, Dennis Hedderich^1^, Dominik Sepp^1^, Bernhard Meyer^2^, Chiara Negwer^2^, Jannis Bodden^1^, Jan Kirschke^1^, Tobias Boeckh-Behrens^1,*^ and, Christian Maegerlein^1,*^

**Corresponding author**: Maximilian Rothe (maximilian.rothe@tum.de)

^1^ Institute of Neuroradiology, School of Medicine and Health, Technical University of Munich, Munich, Germany

^2^ Department of Neurosurgery, School of Medicine and Health, Technical University of Munich, Munich, Germany

**Supplementary Table 1. Overall data**

| **Characteristics** | **Overall** | **Derivo** | **Non-Derivo** |
| --- | --- | --- | --- |
| Number of patients | 104 | 33 | 76 |
| Number of FD | 121 | 35 | 86 |
| Number of aneurysms | 127 | 39 | 90 |
| Number of FD procedures | 112 | 34 | 80 |
| age (years) (mean ± SD) | 55.0 ± 13.4 | 53.3 ± 14.8 | 55.2 ± 12.8 |
| *Sex* | (n=104) | (n=33) | (n=76) |
| Female | 79 (76.0) | 24 (72.7) | 59 (77.6) |
| Male | 25 (24.0) | 9 (27.3) | 17 (22.4) |
| *Presentation* | (n=127) | (n=39) | (n=90) |
| Acute SAH | 11 (8.7) | 2 (5.1) | 10 (11.1) |
| Asymptomatic | 72 (56.7) | 26 (66.7) | 47 (52.2) |
| Recurrence | 19 (15.0) | 3 (7.7) | 17 (18.9) |
| Symptoms other than SAH | 25 (19.7) | 8 (20.5) | 16 (17.8) |
| *Circulation* | (n=127) | (n=39) | (n=90) |
| Anterior | 100 (78.7) | 33 (84.6) | 68 (75.6) |
| Posterior | 27 (21.3) | 6 (15.4) | 22 (24.2) |
| *Location* | (n=127) | (n=39) | (n=90) |
| ICA | 79 (62.2) | 29 (74.4) | 50 (55.6) |
| Basilar artery | 9 (7.1) | 5 (12.8) | 4 (4.4) |
| MCA | 3 (2.4) | 1 (2.6) | 2 (2.2) |
| PCA | 8 (6.3) | 0 (0.0) | 8 (8.9) |
| ACA | 4 (3.1) | 1 (2.6) | 3 (3.3) |
| ACoA | 9 (7.1) | 1 (2.6) | 9 (10.0) |
| PCoA | 4 (3.1) | 1 (2.6) | 4 (4.4) |
| Vertebral artery | 7 (5.5) | 1 (2.6) | 6 (6.7) |
| SCA | 1 (0.8) | 0 (0.0) | 1 (1.1) |
| AICA | 1 (0.8) | 0 (0.0) | 1 (1.1) |
| PICA | 2 (1.6) | 0 (0.0) | 2 (2.2) |
| *Morphology* | (n=127) | (n=39) | (n=90) |
| Saccular | 99 (78.0) | 30 (76.9) | 70 (77.8) |
| Fusiform | 18 (14.2) | 8 (20.5) | 10 (11.1) |
| Blister-like | 4 (3.1) | 1 (2.6) | 4 (4.4) |
| Dissecting | 5 (3.9) | 0 (0.0) | 5 (5.6) |
| Mycotic | 1 (0.8) | 0 (0.0) | 1 (1.1) |
| *Size* | (n=127) | (n=39) | (n=90) |
| Small (<10 mm) | 100 (78.7) | 23 (59.0) | 79 (87.8) |
| Large (10–<25 mm) | 25 (19.7) | 15 (38.5) | 10 (11.1) |
| Giant (≥25 mm) | 2 (1.6) | 1 (2.6) | 1 (1.1) |
| Neck width (mm) (mean ± SD) | 5.85 ± 6.43 | 8.65 ± 9.86 | 4.58 ± 3.51 |
| Ratio aneurysm height / distal vessel diameter (mean ± SD) | 3.01 ± 1.78 | 2.89 ± 1.59 | 3.08 ± 1.92 |
| *FD diameter* | (n=121) | (n=35) | (n=86) |
| Nominal diameter (mm) (median [IQR]) | 4.0 (3.0-4.5) | 4.5 (4.0-5.0) | 4.0 (3.0-4.0) |
| PSVR (mean ± SD) | 1.01 ± 0.21 | 1.09 ± 0.18 | 0.98 ± 0.21 |
| DSVR (mean ± SD) | 1.19 ± 0.26 | 1.22 ± 0.21 | 1.18 ± 0.28 |
| *ICA-tortuosity* | (n=87) | (n=28) | (n=59) |
| Type Ia | 27 (31.0) | 7 (25.0) | 20 (33.9) |
| Type Ib | 25 (28.7) | 10 (35.7) | 15 (25.4) |
| Type II | 13 (14.9) | 3 (10.7) | 10 (16.9) |
| Type III | 12 (13.8) | 5 (17.9) | 7 (11.9) |
| Type IV | 10 (11.5) | 3 (10.7) | 7 (11.9) |
| Distance d (mm) (mean ± SD) | 4.3 ± 4.4 | 4.4 ± 4.6 | 4.2 ± 4.3 |
| *Posterior* genu angle (°) (mean ± SD) | 84.5 ± 39.9 | 82.5 ± 41.2 | 85.4 ± 39.6 |
| *Anterior* genu angle (°) (mean ± SD) | 36.4 ± 15.4 | 37.3 ± 16.6 | 36.1 ± 14.9 |
| d/AP (mean ± SD) | 0.0525 ± 0.0644 | 0.0534 ± 0.0638 | 0.0520 ± 0.0653 |
| *Surface modification* | (n=121) | (n=35) | (n=86) |
| Yes | 48 (39.7) | 6 (17.1) | 42 (48.8) |
| No | 73 (60.3) | 29 (82.9) | 44 (51.2) |
| *DFT technology* | (n=121) | (n=35) | (n=86) |
| Yes | 100 (82.6) | 35 (100.0) | 65 (75.6) |
| No | 21 (17.4) | 0 (0.0) | 21 (24.4) |
| *Thrombus-associated events* | (n=112) | (n=34) | (n=80) |
| Yes | 19 (17.0) | 10 (29.4) | 9 (11.3) |
| No | 93 (83,0) | 24 (70.6) | 71 (88.7) |
| *Neurological deficits* | (n=112) | (n=34) | (n=80) |
| Yes | 13 (11.6) | 7 (20.6) | 6 (7.5) |
| No | 97 (86.6) | 26 (76.5) | 73 (91.3) |
| Not assessable | 2 (1.8) | 1 (2.9) | 1 (1.3) |
| *Neurological outcome* | (n=112) | (n=34) | (n=80) |
| Yes | 4 (3.6) | 2 (5.9) | 2 (2.5) |
| No | 105 (93.8) | 31 (91.2) | 76 (95.0) |
| Not assessable | 3 (2.7) | 1 (2.9) | 2 (2.5) |
| *Occlusion rates (OKM, mOKM)* |  |  |  |
| Time period 1 (3-9 mo.) | 89 (79.5) | 26 (76.5) | 63 (80.8) |
| Time period 2 (up to 24 mo.) | 107 (84.9) | 32 (84.2) | 75 (85.2) |
| Time period 3 (final FU) | 110 (87.3) | 32 (84.2) | 78 (88.6) |
| Mean FU-time (months) (mean ± SD) | 26.0 ± 21.0 | 27.3 ± 22.1 | 25.4 ± 20.6 |
| *DAPT* | (n=104) | (n=33) | (n=76) |
| Length of DAPT (months) (mean ± SD) | 8.1 ± 3.6 | 8.7 ± 4.5 | 7.8 ± 3.0 |
| ASA | 103 (99.0) | 33 (100.0) | 75 (98.7) |
| Prasugrel | 10 (9.6) | 1 (3.0) | 9 (11.8) |
| Clopidogrel | 92 (88.5) | 32 (97.0) | 65 (85.5) |
| Ticagrelor | 1 (1.0) | 0 (0.0) | 1 (1.3) |
| **Improvement of wall apposition** | | | |
| *Overall* | (n=121) | (n=35) | (n=86) |
| Total maneuvers | 43 (35.5) | 21 (60.0) | 22 (25.6) |
| Non-stent maneuvers | 35 (28.9) | 16 (45.7) | 19 (22.1) |
| Stent-based maneuvers | 16 (13.2) | 10 (28.6) | 6 (7.0) |
| *Primary. intraprocedural* | (n=121) | (n=35) | (n=86) |
| Total maneuvers | 38 (31.4) | 18 (51.4) | 20 (23.3) |
| Non-stent maneuvers | 33 (27.3) | 16 (45.7) | 17 (19.8) |
| Proximal | 30 (24.8) | 15 (42.9) | 15 (17.4) |
| Distal | 18 (14.9) | 7 (20.0) | 11 (12.8) |
| Stent-based maneuvers | 10 (8.3) | 7 (20.0) | 3 (3.5) |
| Proximal | 8 (6.6) | 6 (17.1) | 2 (2.3) |
| Distal | 3 (2.5) | 1 (2.9) | 2 (2.3) |
| *Secondary* | (n=121) | (n=35) | (n=86) |
| Total maneuvers | 8 (6.6) | 4 (11.4) | 4 (4.7) |
| Non-stent maneuvers | 4 (3.3) | 1 (2.9) | 3 (3.5) |
| Stent-based maneuvers | 7 (5.8) | 3 (8.6) | 4 (4.7) |
| **Fish-mouthing** |  |  |  |
| *Fish-mouthing-like deformity* | (n=121) | (n=35) | (n=86) |
| Yes | 38 (31.4) | 16 (45.7) | 22 (25.6) |
| No | 75 (62.0) | 16 (45.7) | 59 (68.6) |
| Not assessable | 8 (6.6) | 3 (8.6) | 5 (5.8) |
| *Pre-fish-mouthing* | (n=113) | (n=32) | (n=81) |
| Yes | 22 (19.5) | 10 (31.3) | 12 (14.8) |
| No | 91 (80.5) | 22 (68.8) | 69 (85.2) |
| *Manifest fish-mouthing* | (n=113) | (n=32) | (n=81) |
| Yes | 16 (14.2) | 6 (18.8) | 10 (12.3) |
| No | 97 (85.8) | 26 (81.3) | 71 (87.7) |
| *Grading* | (n=16) | (n=6) | (n=10) |
| Moderate (25-50%) | 10 (62.5) | 3 (50.0) | 7 (70.0) |
| Significant (50-75%) | 4 (25.0) | 1 (16.7) | 3 (30.0) |
| Severe (≥75%) | 2 (12.5) | 2 (33.3) | 0 (0.0) |
| *Anatomical distribution* | (n=38) | (n=16) | (n=22) |
| Proximal | 17 (44.7) | 9 (56.3) | 8 (36.4) |
| Distal | 26 (68.4) | 10 (62.5) | 16 (72.7) |
| *Retreatment?* | (n=38) | (n=16) | (n=22) |
| Yes | 7 (18.4) | 2 (12.5) | 1 (4.5) |
| No | 31 (81.6) | 14 (87.5) | 21 (95.5) |
| *Course* | (n=38) | (n=16) | (n=22) |
| Regressive | 19 (50.0) | 11 (68.8) | 8 (36.4) |
| Constant | 1 (2.6) | 1 (6.3) | 0 (0.0) |
| Progressive | 0 (0.0) | 0 (0.0) | 0 (0.0) |
| Not assessable | 18 (47.4) | 4 (24.9) | 14 (73.6) |
| *Time of occurrence* | (n=38) | (n=16) | (n=22) |
| Periprocedural | 4 (10.5) | 2 (12.5) | 2 (9.1) |
| Time of determination (months) (mean ± SD) | 5.3 ± 2.6 | 5.0 ± 2.8 | 5.6 ± 2.5 |

*All data are presented as n (%) unless specified otherwise.*

*Derivo, DERIVO Embolization Device; FD, Flow diverter; SAH, subarachnoid hemorrhage; ICA, internal carotid artery; MCA, middle cerebral artery; PCA, posterior cerebral artery; ACA, anterior cerebral artery; ACoA, anterior communicating artery; PCoA, posterior communicating artery; SCA, superior cerebellar artery; AICA, anterior inferior cerebellar Artery; PICA, posterior inferior cerebellar Artery; PSVR, proximal stent-to-vessel ratio; DSVR, distal stent-to-vessel ratio; AP, sum of the anterior and posterior ICA knee angles; DFT, drawn filled tubing; OKM, O´Kelly Marotta grading scale; mOKM, modified O´Kelly Marotta grading scale; FU, Follow Up; DAPT, dual antiplatelet therapy; ASA, Acetylsalicylic Acid*

**Supplementary Table 2. Overall data - DED 1 vs. DED 2**

| **Characteristics** | **DED Overall** | **DED 1** | **DED 2** |
| --- | --- | --- | --- |
| Number of patients | 33 | 17 | 16 |
| Number of FD | 35 | 19 | 16 |
| Number of aneurysms | 39 | 20 | 19 |
| Number of FD procedures | 34 | 18 | 16 |
| age (years) (mean ± SD) | 53.3 ± 14.8 | 55.0 ± 12.7 | 51,8 ± 16.5 |
| *Sex* | (n=33) | (n=17) | (n=16) |
| Female | 24 (72.7) | 13 (76.5) | 11 (68.8) |
| Male | 9 (27.3) | 4 (23.5) | 5 (31.3) |
| *Presentation* | (n=39) | (n=20) | (n=19) |
| Acute SAH | 2 (5.1) | 0 (0.0) | 2 (10.5) |
| Asymptomatic | 26 (66.7) | 17 (85.0) | 11 (57.9) |
| Recurrence | 3 (7.7) | 1 (5.0) | 2 (10.5) |
| Symptoms other than SAH | 8 (20.5) | 2 (10.0) | 4 (21.1) |
| *Circulation* | (n=39) | (n=20) | (n=19) |
| Anterior | 33 (84.6) | 18 (90.0) | 15 (78.9) |
| Posterior | 6 (15.4) | 2 (10.0) | 4 (21.1) |
| *Location* | (n=39) | (n=20) | (n=19) |
| ICA | 29 (74.4) | 17 (85.0) | 12 (63.2) |
| Basilar artery | 5 (12.8) | 2 (10.0) | 3 (15.8) |
| MCA | 1 (2.6) | 1 (5.0) | 0 (0.0) |
| PCA | 0 (0.0) | 0 (0.0) | 0 (0.0) |
| ACA | 1 (2.6) | 0 (0.0) | 1 (5.3) |
| ACoA | 1 (2.6) | 0 (0.0) | 1 (5.3) |
| PCoA | 1 (2.6) | 0 (0.0) | 1 (5.3) |
| Vertebral artery | 1 (2.6) | 0 (0.0) | 1 (5.3) |
| SCA | 0 (0.0) | 0 (0.0) | 0 (0.0) |
| AICA | 0 (0.0) | 0 (0.0) | 0 (0.0) |
| PICA | 0 (0.0) | 0 (0.0) | 0 (0.0) |
| *Morphology* | (n=39) | (n=20) | (n=19) |
| Saccular | 30 (76.9) | 19 (95.0) | 11 (57.9) |
| Fusiform | 8 (20.5) | 1 (5.0) | 7 (36.8) |
| Blister-like | 1 (2.6) | 0 (0.0) | 1 (5.3) |
| Dissecting | 0 (0.0) | 0 (0.0) | 0 (0.0) |
| Mycotic | 0 (0.0) | 0 (0.0) | 0 (0.0) |
| *Size* | (n=39) | (n=20) | (n=19) |
| Small (<10 mm) | 23 (59.0) | 10 (50.0) | 13 (68.4) |
| Large (10–<25 mm) | 15 (38.5) | 10 (50.0) | 5 (26.3) |
| Giant (≥25 mm) | 1 (2.6) | 0 (0.0) | 1 (5.3) |
| Neck width (mm) (mean ± SD) | 8.65 ± 9.86 | 7.76 ± 10.02 | 9.59 ± 9.87 |
| Ratio aneurysm height / distal vessel diameter (mean ± SD) | 2.89 ± 1.59 | 2.05 | 3.01 ± 1.67 |
| *FD diameter* | (n=35) | (n=19) | (n=16) |
| Nominal diameter (mm) (median [IQR]) | 4.5 (4.0-5.0) | 4.5 (4.0-5.0) | 5.0 (4.0-6.0) |
| PSVR (mean ± SD) | 1.09 ± 0.18 | 1.05 ± 0.13 | 1.14 ± 0.23 |
| DSVR (mean ± SD) | 1.22 ± 0.21 | 1.18 ± 0.20 | 1.26 ± 0.22 |
| *ICA-tortuosity* | (n=28) | (n=15) | (n=13) |
| Type Ia | 7 (25.0) | 3 (20.0) | 4 (30.8) |
| Type Ib | 10 (35.7) | 5 (33.3) | 5 (38.5) |
| Type II | 3 (10.7) | 1 (6.7) | 2 (15.4) |
| Type III | 5 (17.9) | 4 (26.7) | 1 (7.7) |
| Type IV | 3 (10.7) | 2 (13.3) | 1 (7.7) |
| Distance d (mm) (mean ± SD) | 4.4 ± 4.6 | 4.9 ± 4.5 | 3.9 ± 4.9 |
| *Posterior* genu angle (°) (mean ± SD) | 82.5 ± 41.2 | 75.6 ± 45.1 | 90.6 ± 36.3 |
| *Anterior* genu angle (°) (mean ± SD) | 37.3 ± 16.6 | 40.3 ± 18.5 | 33.7 ± 13.9 |
| d/AP (mean ± SD) | 0.0534 ± 0.0638 | 0.0630 ± 0.0668 | 0.0423 ± 0.0608 |
| *Surface modification* | (n=35) | (n=19) | (n=16) |
| Yes | 6 (17.1) | 0 (0.0) | 6 (37.5) |
| No | 29 (82.9) | 19 (100.0) | 10 (62.5) |
| *DFT technology* | (n=35) | (n=19) | (n=16) |
| Yes | 35 (100.0) | 19 (100.0) | 16 (100.0) |
| No | 0 (0.0) | 0 (0.0) | 0 (0.0) |
| *Thrombus-associated events* | (n=34) | (n=18) | (n=16) |
| Yes | 10 (29.4) | 4 (22.2) | 6 (37.5) |
| No | 24 (70,6) | 14 (77.8) | 10 (62.5) |
| *Neurological deficits* | (n=34) | (n=18) | (n=16) |
| Yes | 7 (20.6) | 4 (22.2) | 3 (18.8) |
| No | 26 (76.5) | 13 (72.2) | 13 (81.3) |
| Not assessable | 1 (2.9) | 1 (5.1) | 0 (0.0) |
| *Neurological outcome* | (n=34) | (n=18) | (n=16) |
| Yes | 2 (5.9) | 2 (11.1) | 0 (0.0) |
| No | 31 (91.2) | 15 (83.3) | 16 (100.0) |
| Not assessable | 1 (2.9) | 1 (5.1) | 0 (0.0) |
| *Occlusion rate (OKM, mOKM)* |  |  |  |
| Time period 1 (3-9 Mo.) | 26 (76.5) | 15 (78.9) | 11 (73.3) |
| Time period 2 (up to 24 Mo.) | 32 (84.2) | 17 (89.5) | 15 (78.9) |
| Time period 3 (final FU) | 32 (84.2) | 17 (89.5) | 15 (78.9) |
| Mean FU-time (months) (mean ± SD) | 27.3 ± 22.1 | 32.5 ± 29.7 | 20.9 ± 12.3 |
| *DAPT* | (n=33) | (n=17) | (n=16) |
| Length of DAPT (months) (mean ± SD) | 8.7 ± 4.5 | 9.3 ± 5.1 | 8.4 ± 3.9 |
| ASA | 33 (100.0) | 17 (100.0) | 16 (100.0) |
| Prasugrel | 1 (3.0) | 1 (5.9) | 0 (0.0) |
| Clopidogrel | 32 (97.0) | 16 (94.1) | 16 (100.0) |
| Ticagrelor | 0 (0.0) | 0 (0.0) | 0 (0.0) |
| **Improvement of wall apposition** | | | |
| *Overall* | (n=35) | (n=19) | (n=16) |
| Total maneuvers | 21 (60.0) | 10 (52.6) | 11 (68.8) |
| Non-stent maneuvers | 16 (45.7) | 6 (31.6) | 10 (62.5) |
| Stent-based maneuvers | 10 (28.6) | 5 (26.3) | 5 (31.3) |
| *Primary. intraprocedural* | (n=35) | (n=19) | (n=16) |
| Total maneuvers | 18 (51.4) | 8 (42.1) | 10 (62.5) |
| Non-stent maneuvers | 16 (45.7) | 6 (31.6) | 10 (62.5) |
| Proximal | 15 (42.9) | 5 (26.3) | 10 (62.5) |
| Distal | 7 (20.0) | 3 (15.8) | 4 (25.0) |
| Stent-based maneuvers | 7 (20.0) | 3 (15.8) | 4 (25.0) |
| Proximal | 6 (17.1) | 3 (15.8) | 3 (18.8) |
| Distal | 1 (2.9) | 0 (0.0) | 1 (6.3) |
| *Secondary* | (n=35) | (n=19) | (n=16) |
| Total maneuvers | 4 (11.4) | 2 (10.5) | 2 (12.5) |
| Non-stent maneuvers | 1 (2.9) | 0 (0.0) | 1 (6.3) |
| Stent-based maneuvers | 3 (8.6) | 2 (10.5) | 1 (6.3) |
| **Fish-mouthing** |  |  |  |
| *Fish-mouthing-like deformity* | (n=35) | (n=19) | (n=16) |
| Yes | 16 (45.7) | 8 (42.1) | 8 (50.0) |
| No | 16 (45.7) | 10 (52.6) | 6 (37.5) |
| Not assessable | 3 (8.6) | 1 (5.3) | 2 (12.5) |
| *Pre-fish-mouthing* | (n=32) | (n=18) | (n=14) |
| Yes | 10 (31.3) | 5 (27.8) | 5 (35.7) |
| No | 22 (68.8) | 13 (72.2) | 9 (64.3) |
| *Manifest fish-mouthing* | (n=32) | (n=18) | (n=14) |
| Yes | 6 (18.8) | 3 (16.7) | 3 (21.4) |
| No | 26 (81.3) | 15 (83.3) | 11 (78.6) |
| *Grading* | (n=6) | (n=3) | (n=3) |
| Moderate (25-50%) | 3 (50.0) | 2 (66.7) | 1 (33.3) |
| Significant (50-75%) | 1 (16.7) | 1 (33.3) | 0 (0.0) |
| Severe (≥75%) | 2 (33.3) | 0 (0.0) | 2 (66.7) |
| *Anatomical distribution?* | (n=16) | (n=8) | (n=8) |
| Proximal | 9 (56.3) | 3 (37.5) | 6 (75.0) |
| Distal | 10 (62.5) | 5 (62.5) | 5 (62.5) |
| *Retreatment?* | (n=16) | (n=8) | (n=8) |
| Yes | 2 (12.5) | 1 (12.5) | 1 (12.5) |
| No | 14 (87.5) | 7 (87.5) | 7 (87.5) |
| *Course* | (n=16) | (n=8) | (n=8) |
| Regredient | 11 (68.8) | 7 (87.5) | 4 (50.0) |
| Constant | 1 (6.3) | 1 (12.5) | 0 (0.0) |
| Progressive | 0 (0.0) | 0 (0.0) | 0 (0.0) |
| Not assessable | 4 (24.9) | 0 (0.0) | 4 (50.0) |
| *Time of occurrence* | (n=16) | (n=8) | (n=8) |
| Periprocedural | 2 (12.5) | 2 (25.0) | 0 (0.0) |
| Time of determination (months) (mean ± SD) | 5.0 ± 2.8 | 4.0 ± 2.8 | 6.0 ± 2.5 |

*All data are presented as n (%) unless specified otherwise.*

*DED, DERIVO Embolization Device; FD, Flow diverter; SAH, subarachnoid hemorrhage; ICA, internal carotid artery; MCA, middle cerebral artery; PCA, posterior cerebral artery; ACA, anterior cerebral artery; ACoA, anterior communicating artery; PCoA, posterior communicating artery; SCA, superior cerebellar artery; AICA, anterior inferior cerebellar Artery; PICA, posterior inferior cerebellar Artery; PSVR, proximal stent-to-vessel ratio; DSVR, distal stent-to-vessel ratio; AP, sum of the anterior and posterior ICA knee angles; DFT, drawn filled tubing; OKM, O´Kelly Marotta grading scale; mOKM, modified O´Kelly Marotta grading scale; FU, Follow Up; DAPT, dual antiplatelet therapy; ASA, Acetylsalicylic Acid*

**Supplementary Table 3. Baseline Characteristics – DED 1 vs. DED 2**

| **Characteristics** | **DED Overall** | **DED 1** | **DED 2** | **P-value** |
| --- | --- | --- | --- | --- |
| Number of patients | 33 | 17 | 16 |  |
| Number of FD | 35 | 19 | 16 |  |
| Number of aneurysms | 39 | 20 | 19 |  |
| Number of FD procedures | 34 | 18 | 16 |  |
| age (years) (mean ± SD) | 53.3 ± 14.8 | 55.0 ± 12.7 | 51,8 ± 16.5 | 0.540 |
| *Sex* | (n=33) | (n=17) | (n=16) | 0.708 |
| Female | 24 (72.7) | 13 (76.5) | 11 (68.8) |  |
| Male | 9 (27.3) | 4 (23.5) | 5 (31.3) |  |
| *Presentation* | (n=39) | (n=20) | (n=19) |  |
| Acute SAH | 2 (5.1) | 0 (0.0) | 2 (10.5) | 0.231 |
| Asymptomatic | 26 (66.7) | 17 (85.0) | 11 (57.9) | 0,082 |
| Recurrence | 3 (7.7) | 1 (5.0) | 2 (10.5) | 0,605 |
| Symptoms other than SAH | 8 (20.5) | 2 (10.0) | 4 (21.1) | 0,445 |
| *Circulation* | (n=39) | (n=20) | (n=19) | 0.408 |
| Anterior | 33 (84.6) | 18 (90.0) | 15 (78.9) |  |
| Posterior | 6 (15.4) | 2 (10.0) | 4 (21.1) |  |
| *Location* | (n=39) | (n=20) | (n=19) |  |
| ICA | 29 (74.4) | 17 (85.0) | 12 (63.2) | 0.155 |
| Basilar artery | 5 (12.8) | 2 (10.0) | 3 (15.8) | 0.661 |
| MCA | 1 (2.6) | 1 (5.0) | 0 (0.0) | 1.000 |
| PCA | 0 (0.0) | 0 (0.0) | 0 (0.0) | - |
| ACA | 1 (2.6) | 0 (0.0) | 1 (5.3) | 0.487 |
| ACoA | 1 (2.6) | 0 (0.0) | 1 (5.3) | 0.487 |
| PCoA | 1 (2.6) | 0 (0.0) | 1 (5.3) | 0.487 |
| Vertebral artery | 1 (2.6) | 0 (0.0) | 1 (5.3) | 0.487 |
| SCA | 0 (0.0) | 0 (0.0) | 0 (0.0) | - |
| AICA | 0 (0.0) | 0 (0.0) | 0 (0.0) | - |
| PICA | 0 (0.0) | 0 (0.0) | 0 (0.0) | - |
| *Morphology* | (n=39) | (n=20) | (n=19) |  |
| Saccular | 30 (76.9) | 19 (95.0) | 11 (57.6) | **0.008** |
| Fusiform | 8 (20.5) | 1 (5.0) | 7 (36.8) | **0.020** |
| Blister-like | 1 (2.6) | 0 (0.0) | 1 (5.3) | 0.487 |
| Dissecting | 0 (0.0) | 0 (0.0) | 0 (0.0) | - |
| Mycotic | 0 (0.0) | 0 (0.0) | 0 (0.0) | - |
| *Size* | (n=39) | (n=20) | (n=19) |  |
| Small (<10 mm) | 23 (59.0) | 10 (10.0) | 13 (68.4) | 0.333 |
| Large (10–<25 mm) | 15 (38.5) | 10 (10.0) | 5 (26.3) | 0.191 |
| Giant (≥25 mm) | 1 (2.6) | 0 (0.0) | 1 (5.3) | 0.487 |
| Neck width (mm) (mean ± SD) | 8.65 ± 9.86 | 7.76 ± 10.02 | 9.59 ± 9.87 | 0.972 |
| Ratio aneurysm height / distal vessel diameter (mean ± SD) | 2.89 ± 1.59 | 2.05 | 3.01 ± 1.67 | 0.611 |
| *Surface modification* | (n=35) | (n=19) | (n=16) | **0.005** |
| Yes | 6 (17.1) | 0 (0.0) | 6 (37.5) |  |
| No | 29 (82.9) | 19 (100.0) | 10 (62.5) |  |
| *DFT technology* | (n=35) | (n=19) | (n=16) | - |
| Yes | 35 (100.0) | 19 (100.0) | 16 (100.0) |  |
| No | 0 (0.0) | 0 (0.0) | 0 (0.0) |  |
| *FD diameter* | (n=35) | (n=19) | (n=16) |  |
| Nominal diameter (median [IQR]) (mm) | 4.5 (4.0-5.0) | 4.5 (4.0-5.0) | 5.0 (4.0-6.0) | 0.144 |
| PSVR (mean ± SD) | 1.09 ± 0.18 | 1.05 ± 0.13 | 1.14 ± 0.23 | 0.271 |
| DSVR (mean ± SD) | 1.22 ± 0.21 | 1.18 ± 0.20 | 1.26 ± 0.22 | 0.595 |

*All data are presented as n (%) unless specified otherwise.*

*DED, DERIVO Embolization Device; FD, Flow diverter; SAH, subarachnoid hemorrhage; ICA, internal carotid artery; MCA, middle cerebral artery; PCA, posterior cerebral artery; ACA, anterior cerebral artery; ACoA, anterior communicating artery; PCoA, posterior communicating artery; SCA, superior cerebellar artery; AICA, anterior inferior cerebellar Artery; PICA, posterior inferior cerebellar Artery; DFT, drawn filled tubing; PSVR, proximal stent-to-vessel ratio; DSVR, distal stent-to-vessel ratio*

**Supplementary Table 4. Fish-mouthing - Incidence, Distribution & Course (DED 1 vs. DED 2)**

| **Variable** | **DED 1 (n=18)** | **DED 2 (n=14)** | **P-value** |
| --- | --- | --- | --- |
| Incidence of fish-mouthing-like deformity | 8/18 (44.4%) | 8/14 (57.1%) | 0.476 |
| Incidence of pre-fish-mouthing | 5/18 (27.8) | 5/14 (35.7) | 0.712 |
| Incidence of manifest fish-mouthing | 3/18 (16.7) | 3/14 (21.4) | 1.000 |
| Severity |  |  | 0.320 |
| Moderate | 2/18 (11.1%) | 1/14 (7.1%) | 0.424 |
| Significant | 1/18 (5.6%) | 0/14 (0.0%) | 1.000 |
| Severe | 0/18 (0.0%) | 2/14 (14.3%) | 0.183 |
| Anatomical distribution of fish-mouthing-like deformity |  |  |  |
| Proximal | 3/18 (16.7%) | 6/14 (42.9%) | 0.132 |
| Distal | 5/18 (27.8%) | 5/14 (35.7%) | 0.712 |
| Retreatment | 1/8 (12.5%) | 1/8 (12.5%) | 1.000 |

*DED, DERIVO Embolization Device*

**Supplementary Table 5. Mechanical improvement of wall apposition (DED 1 vs. DED 2)**

| **Variable** | **DED 1 (n=19)** | **DED 2 (n=16)** | **P-value** |
| --- | --- | --- | --- |
| Overall maneuvers | 10/19 (52.6%) | 11/16 (68.8%) | 0.491 |
| Primary maneuvers | 8/19 (42.1) | 10/16 (62.5%) | 0.315 |
| Proximal Stent | 3/19 (15.8%) | 3/16 (18.8%) | 1.000 |
| Proximal Non-Stent | 5/19 (26.3%) | 10/16 (62.5%) | **0.044** |
| Distal Stent | 0/19 (0.0%) | 1/16 (6.3%) | 0.457 |
| Distal Non-Stent | 3/19 (15.8%) | 4/16 (25.0%) | 0.677 |
| Secondary maneuvers | 2/19 (10.5%) | 2/16 (12.5%) | 1.000 |

*DED, DERIVO Embolization Device; Non-Stent: Balloon angioplasty, microcatheter, distal access catheter, or remodeling device utilization*

**Supplementary Table 6. Fish-mouthing - Incidence, Distribution & Course (first generation p64 vs. p64 MW)**

| **Variable** | **p64 first generation (n=15)** | **p64 MW (n=33)** | **P-value** |
| --- | --- | --- | --- |
| Incidence of fish-mouthing-like deformity | 4/15 (26.7%) | 10/33 (30.3%) | 1.000 |
| Incidence of pre-fish-mouthing | 2/15 (13.3%) | 3/33 (9.1%) | 0.642 |
| Incidence of manifest fish-mouthing | 2/15 (13.3%) | 7/33 (21.2%) | 0.700 |
| Severity |  |  | 0.603 |
| Moderate | 2/15 (13.3%) | 5/33 (15.2%) | 1.000 |
| Significant | 0/15 (0.0%) | 2/33 (6.1%) | 0.544 |
| Severe | 0/15 (0.0%) | 0/33 (0.0%) | 1.000 |
| Anatomical distribution of fish-mouthing-like deformity |  |  |  |
| Proximal | 0/15 (0.0%) | 4/33 (12.1%) | 0.294 |
| Distal | 4/15 (26.7%) | 8/33 (24.2%) | 1.000 |
| Retreatment | 0/4 (0.0%) | 1/10 (10.0%) | 1.000 |

**Supplementary Table 7. Mechanical improvement of wall apposition (first generation p64 vs. p64 MW)**

| **Variable** | **p64 first generation (n=15)** | **p64 MW (n=35)** | **P-value** |
| --- | --- | --- | --- |
| Overall maneuvers | 3/15 (20.0%) | 15/35 (42.9%) | 0.123 |
| Primary maneuvers | 3/15 (20.0%) | 14/35 (40.0%) | 0.171 |
| Proximal Stent | 1/15 (6.7%) | 0/35 (0.0%) | 0.300 |
| Proximal Non-Stent | 1/15 (6.7%) | 12/35 (34.3%) | 0.076 |
| Distal Stent | 1/15 (6.7%) | 0/35 (0.0%) | 0.300 |
| Distal Non-Stent | 1/15 (6.7%) | 8/35 (22.9%) | 0.247 |
| Secondary maneuvers | 1/15 (6.7%) | 2/35 (5.7%) | 1.000 |

*Non-Stent: Balloon angioplasty, microcatheter, distal access catheter, or remodeling device utilization*

**Supplementary Table 8. Fish-mouthing - Incidence, Distribution & Course (Silk vs. Silk vista)**

| **Variable** | **Silk (n=5)** | **Silk vista (n=6)** | **P-value** |
| --- | --- | --- | --- |
| Incidence of fish-mouthing-like deformity | 1/5 (20.0%) | 1/6 (16.7%) | 1.000 |
| Incidence of pre-fish-mouthing | 1/5 (20.0%) | 0/6 (0.0%) | 0.455 |
| Incidence of manifest fish-mouthing | 0/5 (0.0%) | 1/6 (16.7%) | 1.000 |
| Severity |  |  | 1.000 |
| Moderate | 0/5 (0.0%) | 0/6 (0.0%) | 1.000 |
| Significant | 0/5 (0.0%) | 1/6 (16.7%) | 1.000 |
| Severe | 0/5 (0.0%) | 0/6 (0.0%) | 1.000 |
| Anatomical distribution of fish-mouthing-like deformity |  |  |  |
| Proximal | 0/5 (0.0%) | 1/6 (16.7%) | 1.000 |
| Distal | 1/5 (20.0%) | 0/6 (0.0%) | 0.455 |
| Retreatment | 0/1 (%) | 0/1 (%) | 1.000 |

**Supplementary Table 9. Mechanical improvement of wall apposition (Silk vs. Silk vista)**

| **Variable** | **Silk (n=5)** | **Silk vista (n=6)** | **P-value** |
| --- | --- | --- | --- |
| Overall maneuvers | 2/5 (40.0%) | 0/6 (0.0%) | 0.182 |
| Primary maneuvers | 2/5 (40.0%) | 0/6 (0.0%) | 0.182 |
| Proximal Stent | 0/5 (0.0%) | 0/6 (0.0%) | 1.000 |
| Proximal Non-Stent | 2/5 (40.0%) | 0/6 (0.0%) | 0.182 |
| Distal Stent | 0/5 (0.0%) | 0/6 (0.0%) | 1.000 |
| Distal Non-Stent | 2/5 (40.0%) | 0/6 (0.0%) | 0.182 |
| Secondary maneuvers | 0/5 (0.0%) | 0/6 (0.0%) | 1.000 |

*Non-Stent: Balloon angioplasty, microcatheter, distal access catheter, or remodeling device utilization*
